# Identifying the First Val281L Mutation Causing Nonclassic Congenital Adrenal Hyperplasia in the Central-East Region of Tunisia

**DOI:** 10.7759/cureus.57124

**Published:** 2024-03-28

**Authors:** Ach Taieb, Hayfa Farid, Oumayma Zarrouk, Fatma Ben Abdessalem, Saoussen Layouni

**Affiliations:** 1 Endocrinology, Farhat Hached University Hospital, Sousse, TUN; 2 Physical Medicine and Rehabilitation, Sahloul University Hospital, Sousse, TUN

**Keywords:** 17-hydroxy-progesterone (17-ohp), adrenal gland, infertility, hirsutism, 21-hydroxylase deficiency

## Abstract

Nonclassic congenital adrenal hyperplasia (NCAH) is a genetic disorder characterized by mutations in the genes encoding enzymes involved in cortisol production, most commonly the 21-hydroxylase enzyme. Unlike classic congenital adrenal hyperplasia (CAH), NCAH typically presents later in life with milder symptoms. The diagnosis of NCAH can be challenging due to its nonspecific symptoms and variable presentation. Early detection is crucial for timely intervention and management, particularly in families with a history of the condition. We report a case of NCAH in a patient from the Central-East Region of Tunisia, in whom the subsequent genetic testing revealed a Val281Leu (V281L) mutation in the CYP21A2 gene.

A 26-year-old female presented with facial hirsutism and irregular menstrual cycles. Physical examination revealed mild hirsutism and laboratory tests showed elevated levels of testosterone and 17-hydroxyprogesterone (17-OHP). A provisional diagnosis of NCAH was made, subsequently confirmed by an adrenocorticotropic hormone (ACTH) stimulation test demonstrating an exaggerated 17-OHP response. Genetic testing revealed heterozygosity for the V281L mutation. Family testing showed the patient's mother to be homozygous and the father heterozygous for the mutation.

This report highlights the importance of recognizing subtle symptoms of NCAH for early diagnosis and management. Genetic testing aids in confirming the diagnosis and identifying carriers within families. Treatment with glucocorticoids aims to suppress adrenal androgen production and manage symptoms. Regular follow-up is essential to monitor treatment response and adjust medication as needed. NCAH can present with subtle symptoms, necessitating a high index of suspicion for a proper diagnosis. Genetic testing plays a crucial role in confirming the diagnosis and identifying carriers within families. Early intervention and regular follow-up improve outcomes in affected individuals. This report also underscores the significance of genetic testing in the management of NCAH and highlights the need for increased awareness about this condition among healthcare providers.

## Introduction

Nonclassic congenital adrenal hyperplasia (NCAH) is a genetic disorder characterized by a deficiency in enzymes involved in the production of cortisol [1]. Specifically, NCAH results from mutations in the genes, most commonly the 21-hydroxylase enzyme, with a reported prevalence of 1 in 1000 [1]. Unlike classic congenital adrenal hyperplasia (CAH), which presents in infancy with severe symptoms such as salt wasting and ambiguous genitalia, NCAH typically manifests later in childhood or during adolescence, and its symptoms are milder [2]. Individuals with NCAH may experience symptoms such as irregular menstrual periods, hirsutism, acne, and fertility issues in females, while males may exhibit symptoms such as early puberty and reduced fertility [2].

The diagnostic challenge in NCAH stems from several factors. Firstly, the symptoms of NCAH can be subtle and nonspecific, often overlapping with those of other conditions or even normal variations. Secondly, there is variability in the severity and presentation of NCAH among affected individuals, making it challenging to establish clear diagnostic criteria applicable to all cases [3]. Diagnosing NCAH even in cases of mild hirsutism is crucial for identifying carriers within the family, thereby enabling early intervention and management, preventing potential complications, and facilitating screening and management of other family members [4]. Once NCAH is identified in one family member, it is essential to screen other family members for the condition. This proactive approach allows for the early detection and management of NCAH in affected individuals, reducing the risk of complications and improving overall health outcomes within the family [5]. We report the first case involving the detection of a Val281Leu mutation in a family in the Central-East Region of Tunisia.

## Case presentation

A 26-year-old female with no significant medical history presented to an endocrinologist for the assessment of her facial hirsutism. The patient had experienced menarche at the age of 12 years. She also reported irregular menstrual cycles occurring every two to three months. Her medical history was unremarkable, and she denied any symptoms suggestive of virilization such as voice deepening or clitoromegaly. She was not taking any medications or supplements. Additionally, there was a family history of infertility and hirsutism in her female cousins. Her two parents were first cousins originally from the Central-East Region of Tunisia (Kairouan). Her family history was marked by cardiovascular diseases, hirsutism, and an unexplored case of infertility.

On physical examination, the patient exhibited hirsutism with a Ferriman-Gallwey score of 12, indicating mild excess hair growth. Her blood pressure, body mass index (BMI), and other systemic examinations were within normal limits. Laboratory tests revealed normal levels of morning cortisol, complete blood count, and thyroid-stimulating hormone (TSH). However, total testosterone level was elevated (0.9 ng/mL), along with increased levels of 17-hydroxyprogesterone (17-OHP) (2.4 ng/mL). Other hormone levels were within normal ranges (Table 1).

**Table 1:** Main biological and hormonal assessment TSH: thyroid-stimulating hormone; FT4: free T4; FSH: follicle-stimulating hormone; LH: luteinizing hormone

Samples	Patient value	Normal value
Calcium (mmol/L)	2.45	2.2–2.6
TSH (mUI/mL)	2.5	0.5–4.5
FT4 (pg/L)	13	7–19
FSH (UI/L)	6	5–15
LH (UI/L)	7.6	5–15
Estradiol (UI/L)	86	>40
Cortisol (ng/mL)	189	>180
Testosterone (ng/L)	0.9	<0.6
17 Hydroxy Progesterone (ng/mL)	2.4	<2

Given the clinical presentation of hirsutism, irregular menses, and elevated 17-OHP, a provisional diagnosis of NCAH was considered. The patient underwent an adrenocorticotropic hormone (ACTH) stimulation test, which confirmed the diagnosis by demonstrating an exaggerated rise in 17-OHP levels (from 2.4 ng/mL to 64 ng/dL at 60 minutes post-stimulation). Genetic testing subsequently revealed heterozygosity for a mutation in the CYP21A2 gene, confirming the diagnosis of NCAH.

**Figure 1:**
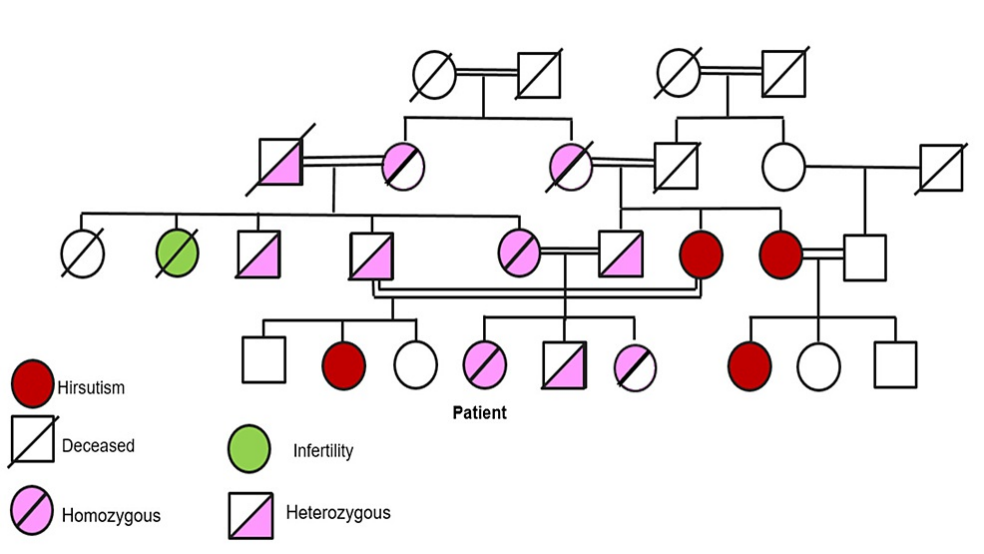
Genetic tree of the patient’s family This genetic tree represents an autosomal recessive inheritance pattern, where both copies of the CYP21A2 gene need to be mutated for the condition to be expressed

The patient and her parents were referred for genetic testing (MLPA confirmed by Sanger sequencing) for NCAH due to 21-OHD with evidence of homozygosity for CYP21A2 mutation: V281Leu. Her mother was also diagnosed as homozygous for the same mutation and her father was heterozygous for the mutation (Figure 1).

The patient was initiated on low-dose glucocorticoid therapy (hydrocortisone 20 mg daily) to suppress adrenal androgen production. She was counseled regarding the chronic nature of NCAH and the importance of regular follow-up for monitoring symptoms and medication adjustments. Additionally, she was advised on cosmetic management of hirsutism, including hair removal techniques such as laser therapy.

At the three-month follow-up visit, the patient reported a reduction in hirsutism and improved menstrual regularity. Repeat laboratory tests showed normalization of 17-OHP levels. The dose of hydrocortisone was adjusted based on clinical response and hormonal parameters.

## Discussion

Hirsutism, characterized by excessive hair growth in a male pattern distribution in females, is a clinical manifestation of androgen excess that often prompts patients to seek medical evaluation due to cosmetic concerns and psychological distress. While hirsutism is commonly associated with polycystic ovary syndrome (PCOS), clinicians must adopt a systematic approach to its diagnosis, ruling out other potential etiologies, including rare disorders such as NCAH [4]. One of the challenges in managing hirsutism is distinguishing between various underlying causes. While PCOS is the most common endocrine disorder associated with hirsutism, accounting for approximately 70-80% of cases, other conditions such as NCAH, Cushing's syndrome, hyperprolactinemia, and androgen-secreting tumors must also be considered in the differential diagnosis [1]. Failure to identify the correct etiology may result in inadequate treatment and persistent symptoms.

NCAH is an autosomal recessive disorder caused by mutations in the 21-hydroxylase enzyme gene (CYP21A2), leading to impaired cortisol synthesis and subsequent excess adrenal androgen production. Even though it is less common than PCOS, NCAH should be considered, particularly in patients with atypical features such as early-onset hirsutism, menstrual irregularities, and a family history of adrenal disorders [2,4]. The prevalence of NCAH ranges between 1 in 1000 and 1 in 100 globally [1]. Additionally, there seems to be a high frequency of heterozygotes, with carriers remaining unidentified, necessitating genotyping for accurate diagnosis [5].

While ACTH-stimulated 17-OHP is commonly utilized for diagnosis, its predictive value is hindered by overlapping concentrations. In this context, 17-OHP levels aid in diagnosing hyperandrogenism and carrier states but fail to distinguish the non-classical form of 21-hydroxylase deficiency, resulting in a wide range of phenotypic expressions [6]. Moreover, the frequency of NCAH appears to be elevated in certain populations, such as in patients with PCOS. Consequently, it is postulated that the prevalence of silent heterozygotes is significant, potentially leading to variable phenotypes in future generations. Given these findings, genetic screening is strongly recommended for newborns and affected families to enable early detection and appropriate management [7].

The identification of a Val281Leu mutation causing NCAH in the Central-East region of Tunisia underscores the importance of understanding the genetic basis of rare endocrine disorders within specific populations. NCAH is primarily caused by mutations in the CYP21A2 gene, which encodes the 21-hydroxylase enzyme responsible for cortisol synthesis in the adrenal glands. The Val281Leu mutation is one of the most common variants associated with NCAH worldwide. Tunisia, like many other countries in North Africa and the Middle East, has a relatively high prevalence of consanguinity, which contributes to the increased incidence of autosomal recessive disorders within these populations. Consanguineous marriages elevate the risk of inheriting identical recessive alleles, including those associated with NCAH [8]. Hence, identifying prevalent mutations such as Val281Leu is crucial for genetic screening and counseling efforts aimed at reducing the burden of inherited disorders in affected families [8,9].

The Central-East region of Tunisia has been identified as a hotspot for certain genetic disorders due to historical migration patterns and genetic isolation. Studies focusing on the genetic landscape of this region have revealed a higher frequency of specific mutations associated with various inherited conditions [9]. The identification of the Val281Leu mutation causing NCAH in this region further highlights the need for targeted genetic screening programs and increased awareness among healthcare providers and the general population [8,9].

Understanding the molecular basis of NCAH mutations, such as Val281Leu, provides insights into the pathophysiology of the disorder and informs diagnostic approaches and therapeutic interventions. Patients carrying the Val281Leu mutation may exhibit variable clinical presentations, ranging from mild hirsutism and menstrual irregularities to more severe manifestations of androgen excess [10]. Therefore, genetic testing plays a crucial role in confirming the diagnosis of NCAH, particularly in cases where clinical features overlap with other endocrine disorders such as PCOS [11]. 

The missense mutation p.Val281Leu leads to a reduction in enzyme activity by 20-50% in NCAH [8,10,11]. It has been observed that mutations in the CYP21A2 gene, particularly the p.Val281Leu variant, increase the likelihood of hyperandrogenism, as compared to carriers of severe mutations [8]. Moreover, the identification of specific mutations associated with NCAH facilitates genotype-phenotype correlations and may influence treatment decisions and long-term management strategies. Patients harboring the Val281Leu mutation may benefit from early initiation of glucocorticoid therapy to suppress adrenal androgen production and alleviate symptoms of hyperandrogenism [1,3].

Early recognition and diagnosis of NCAH are essential to initiate appropriate treatment and prevent long-term complications associated with androgen excess, such as infertility, metabolic disturbances, and psychological sequelae. A comprehensive evaluation of patients presenting with hirsutism should include a thorough medical history, physical examination, hormonal assays, and, when indicated, genetic testing [1,2]. Furthermore, assessing the family history for similar conditions or consanguinity can provide valuable insights into the likelihood of inherited disorders and guide appropriate management strategies.

## Conclusions

This case report highlights the importance of a thorough diagnostic evaluation in young females presenting with hirsutism to identify underlying endocrine disorders such as NCAH. Early recognition and management of NCAH are crucial to alleviate symptoms, prevent long-term complications, and improve the quality of life in affected individuals. Additionally, this report underscores the need for multidisciplinary collaboration between endocrinologists, gynecologists, and dermatologists in the comprehensive care of patients with hyperandrogenism and associated manifestations.

## References

[1] New MI (2006). Extensive clinical experience: nonclassical 21-hydroxylase deficiency. J Clin Endocrinol Metab.

[2] Witchel S, Azziz R (2010). Nonclassic congenital adrenal hyperplasia. Int J Pediatr Endocrinol.

[3] Dewailly D, Vantyghem-Haudiquet MC, Sainsard C (1986). Clinical and biological phenotypes in late-onset 21-hydroxylase deficiency. J Clin Endocrinol Metab.

[4] Escobar-Morreale HF, San Millán JL, Smith RR, Sancho J, Witchel SF (1999). The presence of the 21-hydroxylase deficiency carrier status in hirsute women: phenotype-genotype correlations. Fertil Steril.

[5] Rabbani B, Mahdieh N, Ashtiani MT (2012). Mutation analysis of the CYP21A2 gene in the Iranian population. Genet Test Mol Biomarkers.

[6] Ghizzoni L, Cappa M, Vottero A (2011). Relationship of CYP21A2 genotype and serum 17-hydroxyprogesterone and cortisol levels in a large cohort of Italian children with premature pubarche. Eur J Endocrinol.

[7] New MI, Lorenzen F, Lerner AJ (1983). Genotyping steroid 21-hydroxylase deficiency: hormonal reference data. J Clin Endocrinol Metab.

[8] Wu DA, Chung BC (1991). Mutations of P450c21 (steroid 21-hydroxylase) at Cys428, Val281, and Ser268 result in complete, partial, or no loss of enzymatic activity, respectively. J Clin Invest.

[9] Kharrat M, Tardy V, M'Rad R (2004). Molecular genetic analysis of Tunisian patients with a classic form of 21-hydroxylase deficiency: identification of four novel mutations and high prevalence of Q318X mutation. J Clin Endocrinol Metab.

[10] Haider S, Islam B, D'Atri V (2013). Structure-phenotype correlations of human CYP21A2 mutations in congenital adrenal hyperplasia. Proc Natl Acad Sci U S A.

[11] Neocleous V, Shammas C, Phedonos AA, Phylactou LA, Skordis N (2014). Phenotypic variability of hyperandrogenemia in females heterozygous for CYP21A2 mutations. Indian J Endocrinol Metab.

